# Assessing Video Games to Improve Driving Skills: A Literature Review and Observational Study

**DOI:** 10.2196/games.3274

**Published:** 2014-08-07

**Authors:** Damian Sue, Pradeep Ray, Amir Talaei-Khoei, Jitendra Jonnagaddala, Suchada Vichitvanichphong

**Affiliations:** ^1^Asia-Pacific Ubiquitous Healthcare Research Centre (APuHC)University of New South WalesSydneyAustralia; ^2^Lab for Agile Information SystemsSchool of Systems, Management, and LeadershipUniversity of Technology, SydneySydneyAustralia; ^3^Research Centre for Human Centered Technology Design (HCTD)University of Technology, SydneySydneyAustralia; ^4^Faculty of Arts and BusinessUniversity of the Sunshine CoastMaroochydoreAustralia

**Keywords:** video games, driving, motor skills, health, coordination

## Abstract

**Background:**

For individuals, especially older adults, playing video games is a promising tool for improving their driving skills. The ease of use, wide availability, and interactivity of gaming consoles make them an attractive simulation tool.

**Objective:**

The objective of this study was to look at the feasibility and effects of installing video game consoles in the homes of individuals looking to improve their driving skills.

**Methods:**

A systematic literature review was conducted to assess the effect of playing video games on improving driving skills. An observatory study was performed to evaluate the feasibility of using an Xbox 360 Kinect console for improving driving skills.

**Results:**

Twenty–nine articles, which discuss the implementation of video games in improving driving skills were found in literature. On our study, it was found the Xbox 360 with Kinect is capable of improving physical and mental activities. Xbox Video games were introduced to engage players in physical, visual and cognitive activities including endurance, postural sway, reaction time, eyesight, eye movement, attention and concentration, difficulties with orientation, and semantic fluency. However, manual dexterity, visuo-spatial perception and binocular vision could not be addressed by these games. It was observed that Xbox Kinect (by incorporating Kinect sensor facilities) combines physical, visual and cognitive engagement of players. These results were consistent with those from the literature review.

**Conclusions:**

From the research that has been carried out, we can conclude that video game consoles are a viable solution for improving user’s physical and mental state. In future we propose to carry a thorough evaluation of the effects of video games on driving skills in elderly people.

##  Introduction

Aging and the lack of physical activity reduce cognitive abilities, as well as physical stamina, which in turn impacts a person’s driving ability [1,2]. In many countries, especially in those with poor public transport, the ability to drive is essential. Without the ability to drive many activities such as visiting health care professionals, socializing, and purchasing groceries could be laborious. This reduces independence and the standard of living of people, thereby leading to increased health risks like reduced cognition and illness [3]. Studies have shown that the most rapidly growing segment of the driving population comprises of people over the age of 65 years [1]. With a larger number of those in the aging population still continuing to drive, it is vital to ensure that their driving skills are maintained. This is essential not only in improving their quality of life, but also to reduce the number of on-road accidents. Studies have shown that older drivers have a higher crash rate per distance travelled, with an increased risk of injury or death in the event of a car crash [4]. It has been shown that continued physical and mental training reduces the degradation of cognitive and physical functions, which are essential for driving [4-7]. Several activities to maintain physical and mental shape include regular physiotherapy, physical workout at the gym, and even playing puzzle games such as crossword. However these activities can be inconvenient to attend and could be boring for few individuals. This has been proven as studies show a 50% drop-out rate from exercise programs within the first 6 months [8]. Therefore, what is needed, is a more cost effective method of engaging individuals in exercise.

Vichitvanichphong et al [9,10] have observed that amongst various technologies adopted for aiding with the independent living of the elderly, games provide the most training to maintain their skills. This is attributed to the interactive nature of games, which thereby empowers them for better daily living. As explained above, one of the important skills for independent living is driving. The advantage that console games have is the ability to engage people in physical activity from the leisure of home while providing an interactive experience. It is also a cost effective method of providing interactivity with other individuals due to accessible, feasibility of set up in multiple homes, and provision of an intuitive feedback without the need to engage medical practitioners on a one-to-one basis. Not only do games improve physical states, but they also affect one’s cognition. With a vast selection of commercially available games, medical professionals could use a wide range of games as part of a daily training program to equip individuals with essential driving skills [11].

Earlier game consoles such as the Super Nintendo Entertainment System and the Nintendo GameCube required a wired controller for players to interact. There is evidence that even these games improved the cognition of players. Recently a newer wave of motion controlled consoles dominates the market. These not only improve the cognition of players, but also the affect their physical state by enabling the use of interactive gestures from the hand and body. Such technology has great potential for improving global health care. The Nintendo Wii released in 2006 has often been credited as the primary console initiating this revolution [11]. Since then several other commercial somatosensory gaming consoles have also entered the market. They include the PlayStation Eye and the Xbox 360 Kinect released in 2010. Xbox Kinect being physically, visually and cognitively engaging has been identified as an appropriate game console for the purpose of this study. To determine the potential and effectiveness of gaming on improving a person’s driving ability, a systematic review was conducted followed by an observatory study where the use of Xbox 360 Kinect games were evaluated.

##  Methods

### Systematic Literature Review

To assess the effect of video games on driving skills, a systematic literature review was conducted to identify the most recent research related to the topic. The review was conducted over 4 months by searching various online databases including Google Scholar, PubMed, Scirus, Institute of Electrical and Electronics Engineers (IEEE) Xplore, Springerlink, ScienceDirect, and Wiley InterScience. The objective was to eliminate all papers that were not relevant to the topic: Video Gaming for Improving Driving Skills. All papers reviewed were written in English and published between the year 2000 and 2012.

In terms of definitions, the topic Video Games included all forms of electronic games including console, computer, motion controlled, and the traditional gamepad/joystick controlled games. Driving skills included cognitive functions, physical ability, and reaction speed. Hence the first step was to identify synonyms for terms “video game” and “driving”. Once a list of synonyms had been compiled, the search phrase was created ("Video Game" OR "Computer Game" OR "exergame" OR "interactive game" OR "XBOX" OR "Wii" OR "PS2" OR "PS3" AND "car" AND "driving"). The word driving was then replaced with other words relating to motor skills which were speed control, lane change, lane observance, head check, shoulder check, lane deviations, touching lane, centerline, parallel parking, staring, pulling curb, signal, indication, turn, grasp wheel, turn wheel, wrong lane, gas, clutch, and seatbelt (ie, "Video Game" OR "Computer Game" OR "exergame" OR "interactive game" OR "XBOX" OR "Wii" OR "PS2" OR "PS3" AND "car " AND "speed control"). These phrases (21 in total) were then entered into each database and all relevant published papers found were screened first by title, then by abstract, and finally by full text. Any repeated articles in the search were excluded.

**Figure 1:**
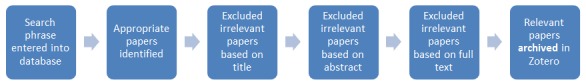
Article selection process for systematic literature review.

The methodology is illustrated in Figure 1. As the review was to look for the empirical evaluation of using gaming, to improve driving of the elderly, any published paper that did not have empirical results was excluded. Once the relevant papers were found they were archived using the reference management software Zotero 4.0, released in 2013 from Roy Rosenzweig Center for History and New Media. One limitation of this review was that the quality of papers, containing the relevant search terms was not assessed. This was due to the limited number of relevant papers. Therefore, all articles found were included.

On reviewing the shortlisted papers it was determined that video games could potentially improve driving skills both in terms of cognition and physical state. It was further determined that there were three major commercial motion controlled gaming consoles currently in the market that could potentially improve the skills of the drivers (Xbox 360 Kinect, Nintendo Wii, and Playstation Eye on the PlayStation 3 system). It was also determined that of the three consoles, Xbox 360 Kinect was the only console that did not require hand-held controllers and also had the ability to track lower body movement. Thus an evaluation of the Xbox 360 Kinect system was conducted to assess how effective it would be.

### Self-Observatory Study: Evaluation of Xbox 360 Kinect Games

An Xbox Kinect System was purchased and tested for its benefits on improving driving skills by three researchers aged between 22 to 40 years. Several games including Kinect Sports, Just Dance 3, Kinect Adventures, Dr Kawashima's Body and Brain Exercises, and Forza Motorsports 4 were purchased for the study. These games were selected on consultation form a panel of experts in order to relate the physical, visual and cognitive functional skills from the game to actual driving skills. Also these games were fun, more enjoyable and served as a cheap alternative to exercise functional abilities. These games were then tested by the researchers between February to July 2012, during which presentations were carried out in small groups of five to ten researchers, where the capabilities of the console were demonstrated. Further discussions were held, wherein ideas and thoughts on the advantages and disadvantages of using the Xbox Kinect system to improve motor skills were discussed.

## Results

### Overview

The results of the literature review are illustrated in Table 1. In summary 150 papers were found to satisfy the criteria based on the abstracts’ relevance to the topic with 78 papers being repeated. Of these, 29 papers were shortlisted based on the full text’s relevance to the topic [12-40]. It was found that the Xbox 360 Kinect had several useful features including various levels of difficulty, improvements in memory, multiple games, reports on progress, entire body training, multiplayer, connecting peripherals including driving wheel and pedals, Internet connectivity, and a freely available software development kit (SDK). Though some of these features may be available in other consoles such as Nintendo Wii and PlayStation 3. The Xbox Kinect, unlike the other systems, need not have sensors attached to the player, thereby providing a more realistic experience.

**Table 1:** Results from systematic review.

Database	Number of results	Number of articles by titles	Number of articles by abstract	Number of repeated articles	Number of articles by full text
Google Scholar	2195	146	78	32	13
SpringerLink	684	74	38	23	7
Wiley InterScience	536	10	2	11	1
ScienceDirect	551	40	11	9	2
Scirus	226	19	6	3	2
IEEE Xplore	47	17	9	6	3
PubMed	46	13	6	4	1
Total	4285	319	150	78	29

### Increasing Level of Difficulty

Several games have varying levels of difficulty, which provide an opportunity for less active players to gradually improve their abilities, rather than being disheartened when attempting a difficult level initially. Games such as Just Dance 3 have two modes, namely the ‘Easy’ and ‘Normal’ modes. The former mode trains only the upper body while the latter mode requires the use of the entire body including the legs and the hip. Another example is Kinect Sports which has 4 levels of difficulty; beginner, amateur, professional, and champion. On our evaluation, the beginner mode was found to be extremely easy and the amateur mode, in our opinion, was sufficiently adequate for keeping the players active.

### Improved Memory

On several games, the player is trained on improving their memory. This could help improve the player’s response time, thereby serving as a crucial skill in identifying signs such as traffic lights and speed limits while driving. The game, Just Dance 3 requires the player to memorize a certain pattern of poses before executing them on the gameplay. Brain and Body Exercises which tests various cognitive functions including math, logic, and memory.

### Multiple Games

The Xbox 360 has a vast array of games, all of which train users at different skills. In our discussions, it was suggested that professionals such as physiotherapists could outline a training program on gaming to ensure the testing for all driving skills. For example Dr Kawashima's Body and Brain Exercises was evaluated for training the cognitive aspects required for driving with its mini games on logic, mathematics, and memory, combined with limited hand eye coordination training. Kinect Sports was found to be better for physical training, with an array of different sports such as soccer, boxing, table tennis, and bowling. Even within Kinect Sports, there were various mini-games, based on the various sports that trained specific muscle movements such as goalkeeping. The physiotherapists involved in our observational study, suggested 10 minutes on table tennis to improve hand eye coordination, followed by 5 minutes of soccer to train the legs, followed by 10 minutes of memory games for mental exercises.

### Reports and Feedback

Most of the games provided a report at the completion of the game. This feature could motivate players and also be used by medical practitioners to monitor their progress. In Just Dance 3, players are rated on their timing and accuracy, with the gameplay choreography. This report could be compared with that of others worldwide. Another example would be Dr Kawashima's Body and Brain Exercises that measured the score over 3 games and provided a brain age score which again could be compared with the player’s previous scores. Though mostly advantageous, this could be a disadvantage as poor scores in a few instances demoralize players.

### Motivational Quotes

On completing certain games, motivational quotes that encourage players to improve on their scores or to train daily are provided. The issues with traditional self-training programs involving stretches and weights is the lack of human motivation on improvement or encouraging further participation. This lack leads to individuals failing to complete their exercises. Consequences like this, could lead to decreased training and eventually the deterioration of their driving skills [10]. Kinect Sports encourages players to exceed the average score recorded on the game and Dr Kawashima's Body and Brain Exercises plays congratulatory music and displays images, when players exceed their previous scores.

### Training Entire Body

Kinect unlike traditional gamepad/joystick games, requires players to move the entire body and this could improve their coordination. In the game, Just Dance 3 the objective is to memorize choreographic moves and to repeat them accurately. This exercises the entire body, enabling better flexibility of the limbs along with improved coordination. Kinect sports has multiple games such as tennis, which requires active movements of the trunk and upper limbs and soccer, requiring mostly lower body movements.

### Ability to Carry Weights

The issue was raised about the lack of resistance when playing games using the Kinect system. While testing the game, Kinect Sports, we noted that players could hold weights in their hands while playing games like bowling and tennis. This could drastically increase muscle weight and strength required for driving in the elderly. We tested this by asking the players to hold one kilogram weights while playing Kinect Sports and assessed their feedback. As expected, the feedback was positive and many stated that this seemed to make the gameplay more real. However, certain issues with controlling were found when holding items such as in the game, Forza Motorsport 3 where a wheel had to be handled while driving. These issues were mostly related to ergonomic concerns and are explained in the discussion section.

### Multiplayer

Most of the games in more recent gaming consoles have a multiplayer mode, wherein the player could compete against other player in their living room or online. This feature that promotes competition, could serve as an impetus to use the Xbox for many individuals. Another advantage is that no additional equipment other than the Kinect sensor is required while playing the multiplayer mode as one Kinect sensor tracks up to 4 players simultaneously. Several games offer multiplayer modes such as tennis and boxing on Kinect Sports, which according our players were exhilarating and definitely a lot of fun. Just Dance 3, Dr Kawashima's Body and Brain Exercises, Forza Motorsports 4, and Kinect Adventures also have multiplayer modes and rank the scores of all players.

### Peripherals: Driving Wheel and Pedals

Certain Xbox 360 games such as Forza Motorsports 3 allow the use of driving wheels and pedals in gameplay, which simulates a true driving experience. This could be very effective for improving driving skills in older adults. An issue however, is the additional cost of purchasing the driving wheel and pedals. Unlike more interactive games such as Kinect Sports, simulated driving games cannot be relied on for fitness training.

### Internet Connectivity

All Xbox 360’s can connect to a network router using a cable with a built-in 10/100 Ethernet network adapter. The upgraded console, Xbox 360 Slim also has built in wireless N 2.4 GHz networking which provides the option of Wi-Fi connectivity. With Internet connectivity, the Xbox 360 provides the option for constant monitoring of results from every game played. These results could be used to assess driving capability, amount of usage and areas for improvement. Several games such as Kinect Sports and Just dance 3 upload scores to the user’s profile by default after obtaining an initial confirmation to do so.

### Xbox Kinect SDK

The SDK is freely available and allows for the development of customized applications. This could be used to design simple, and cost-effective programs targeted specifically at improving specific skills required for driving. These programs can be run by connecting the Kinect System to a computer via a universal serial bus (USB) port. This eliminates the need for a Xbox 360 console, however to play the more interactive, cost effective and robust commercially games such as Kinect Sport, the Xbox 360 console is still required.

### Ergonomic Issues

There were few ergonomic issues found while using the Xbox that could increase the risk of injury. The constant flashing of images during gameplay may trigger nausea and seizures, mostly in those with prior history. One researcher succumbed to this and was slightly ill, while playing Kinect Adventures. This is probably due to the result of quick color alterations that have been built into the games for players to pay more attention [41]. However, this could also cause some visual problems. One solution would be the use of sunglasses while playing [42]. Another concern when using the Xbox to exercise is the likelihood of injury while exercising.

### Costs

The cost of the Xbox 360 250GB Kinect console buddle with three games at a major games retailer in January 2012 was $388 (Australian dollar). The cost of games, range from $20 to $150. The cost of purchasing such a console was relatively affordable and is a viable solution to improve driving ability.

##  Discussion

### Principal Findings

According to our findings there are many benefits to using video games to improve driving ability. Aging and a lack of exercise lead to weakening of the mind and body. However, studies [43] have shown that the deterioration of the body and mind can be slowed down by exercising. Few methods of achieving this include physiotherapy sessions and swimming. Although for many people, these activities may be cumbersome to attend. Another option is to exercise at home, training both physically and completing mental challenges such as crossword puzzles from newspapers and magazines. The issue with this approach is that these exercises could get boring for few and they may neglect to complete them.

We also noticed few ergonomic issues in this study. In active games such as Just dance 3, the possibility of injury is high due to the balance required when twisting the body. However, while playing games such as Body and Brain exercises, the probability of getting injured is less. We find that the fitness of the players needed to be improved gradually either by beginning with less active games, or by beginning at a lower difficulty level and progressively increasing difficulty. It was also suggested that a system be designed to ensure emergency services, should someone be injured. As with physical exercise, players should be aware of their abilities and must avoid putting themselves into undue straining. Video game producers should consider physical limitations of aged players and specific ergonomic requirements could be implemented with more user-friendly factors.

### Conclusions

Video Games, with the advantage of being designed for pleasure, also have the potential to increases physical activity. They are portable and can be easily setup at most homes providing a very convenient method of exercise, especially for those with reduced mobility. The results obtained from the evaluation of an Xbox 360 with Kinect suggest that it is a viable solution for improving the user’s physical and mental state. Although there are a few issues with using the Xbox for exercise, such as risk of injury and nausea, steps could be implemented to overcome these barriers. Gaming consoles can be affordable, convenient, and motivational, thereby helping with the reduction of road accidents and increasing the average driving age. Eventually this improves the quality of life especially in older adults.

### Future Work

Following our observatory study, we recommend that further investigations be conducted to find a correlation between gaming and improving driving skills. A promising approach would be to conduct long-term studies to compare the outcome of driving skills in seniors being trained with Xbox Kinect games.

## Abbreviations

SDK: software development kit
